# Establishing the Canadian HIV Women’s Sexual and Reproductive Health Cohort Study (CHIWOS): Operationalizing Community-based Research in a Large National Quantitative Study

**DOI:** 10.1186/s12874-016-0190-7

**Published:** 2016-08-19

**Authors:** Mona Loutfy, Saara Greene, V. Logan Kennedy, Johanna Lewis, Jamie Thomas-Pavanel, Tracey Conway, Alexandra de Pokomandy, Nadia O’Brien, Allison Carter, Wangari Tharao, Valerie Nicholson, Kerrigan Beaver, Danièle Dubuc, Jacqueline Gahagan, Karène Proulx-Boucher, Robert S. Hogg, Angela Kaida, on Behalf of the CHIWOS Research Team, Aranka Anema, Denise Becker, Lori Brotto, Allison Carter, Claudette Cardinal, Guillaume Colley, Erin Ding, Janice Duddy, Nada Gataric, Robert S. Hogg, Terry Howard, Shahab Jabbari, Evin Jones, Mary Kestler, Andrea Langlois, Viviane Lima, Elisa Lloyd-Smith, Melissa Medjuck, Cari Miller, Deborah Money, Valerie Nicholson, Gina Ogilvie, Sophie Patterson, Neora Pick, Eric Roth, Kate Salters, Margarite Sanchez, Jacquie Sas, Paul Sereda, Marcie Summers, Christina Tom, Clara Wang, Kath Webster, Wendy Zhang, Rahma Abdul-Noor, Jonathan Angel, Fatimatou Barry, Greta Bauer, Kerrigan Beaver, Anita Benoit, Breklyn Bertozzi, Sheila Borton, Tammy Bourque, Jason Brophy, Ann Burchell, Allison Carlson, Lynne Cioppa, Jeffrey Cohen, Tracey Conway, Curtis Cooper, Jasmine Cotnam, Janette Cousineau, Marisol Desbiens, Annette Fraleigh, Brenda Gagnier, Claudine Gasingirwa, Saara Greene, Trevor Hart, Shazia Islam, Charu Kaushic, Logan Kennedy, Desiree Kerr, Maxime Kiboyogo, Gladys Kwaramba, Lynne Leonard, Johanna Lewis, Carmen Logie, Shari Margolese, Marvelous Muchenje, Mary (Muthoni) Ndung’u, Kelly O’Brien, Charlene Ouellette, Jeff Powis, Corinna Quan, Janet Raboud, Anita Rachlis, Edward Ralph, Sean Rourke, Sergio Rueda, Roger Sandre, Fiona Smaill, Stephanie Smith, Tsitsi Tigere, Wangari Tharao, Sharon Walmsley, Wendy Wobeser, Jessica Yee, Mark Yudin, Dada Mamvula Bakombo, Jean-Guy Baril, Nora Butler Burke, Pierrette Clément, Janice Dayle, Danièle Dubuc, Mylène Fernet, Danielle Groleau, Aurélie Hot, Marina Klein, Carrie Martin, Lyne Massie, Brigitte Ménard, Nadia O’Brien, Joanne Otis, Doris Peltier, Alie Pierre, Karène Proulx-Boucher, Danielle Rouleau, Édénia Savoie, Cécile Tremblay, Benoit Trottier, Sylvie Trottier, Christos Tsoukas, Jacqueline Gahagan, Catherine Hankins, Renee Masching, Susanna Ogunnaike-Cooke

**Affiliations:** 1 Women’s College Research Institute, Women’s College Hospital, University of Toronto, 76 Grenville St., Room 6415, Toronto, ON Canada M5S 1B2; 2 Department of Medicine and Institute of Health Policy, Management and Evaluation, University of Toronto, Toronto, Ontario Canada; 3 School of Social Work, McMaster University, Hamilton, Ontario Canada; 4 Interdisciplinary Studies Program, York University, Toronto, Ontario Canada; 5 International Community of Women living with HIV, North America (ICWNA) New Brunswick, New Jersey, USA; 6 Department of Family Medicine, McGill University, Montreal, Quebec Canada; 7 Chronic Viral Illness Service, McGill University Health Centre, Montreal, Quebec Canada; 8 Faculty of Health Sciences, Simon Fraser University, Burnaby, British Columbia Canada; 9 British Columbia Centre for Excellence in HIV/AIDS, Vancouver, British Columbia Canada; 10 Women’s Health in Women’s Hands Community Health Centre, Toronto, Ontario Canada; 11 Health Promotion Division, Dalhousie University Halifax, Nova Scotia, Canada

**Keywords:** Women, HIV, CHIWOS, Community-based research, Cohort study, Research methodology

## Abstract

**Background:**

Community-based research has gained increasing recognition in health research over the last two decades. Such participatory research approaches are lauded for their ability to anchor research in lived experiences, ensuring cultural appropriateness, accessing local knowledge, reaching marginalized communities, building capacity, and facilitating research-to-action. While having these positive attributes, the community-based health research literature is predominantly composed of small projects, using qualitative methods, and set within geographically limited communities. Its use in larger health studies, including clinical trials and cohorts, is limited. We present the Canadian HIV Women’s Sexual and Reproductive Health Cohort Study (CHIWOS), a large-scale, multi-site, national, longitudinal quantitative study that has operationalized community-based research in all steps of the research process. Successes, challenges and further considerations are offered.

**Discussion:**

Through the integration of community-based research principles, we have been successful in: facilitating a two-year long formative phase for this study; developing a novel survey instrument with national involvement; training 39 Peer Research Associates (PRAs); offering ongoing comprehensive support to PRAs; and engaging in an ongoing iterative community-based research process. Our community-based research approach within CHIWOS demanded that we be cognizant of challenges managing a large national team, inherent power imbalances and challenges with communication, compensation and volunteering considerations, and extensive delays in institutional processes. It is important to consider the iterative nature of community-based research and to work through tensions that emerge given the diverse perspectives of numerous team members.

**Conclusions:**

Community-based research, as an approach to large-scale quantitative health research projects, is an increasingly viable methodological option. Community-based research has several advantages that go hand-in-hand with its obstacles. We offer guidance on implementing this approach, such that the process can be better planned and result in success.

## Background

As an approach to research, community-based research (CBR) focuses on acknowledging the inequities that exist between researchers, participants and community members [1, 2]. It ensures that the research question, method, design and the utilization of the data are guided by the community [3–8]. In CBR, researchers and community members engage in partnerships that equally value lived experience and academic expertise in an attempt to minimize inequities [1]. Importantly, CBR attempts to ensure that results reflect the community’s vision of change [1, 9, 10]. The relevance of CBR findings in health research today is established through these partnerships and the creation of research that is translatable given the collaborative process [1, 11]. Despite these benefits, uptake has been stalled because of struggles to determine how to include the iterative nature of CBR into the rigorous process of health research [1, 2, 12]. When used, CBR approaches are most commonly adopted in relatively small studies, conducted in a limited geographic area [2, 13–15]. The limited uptake is despite the fact that CBR has been found to improve research recruitment, response rates and retention [16], particularly among minority groups [17, 18]; to improve community investment in research; and to increase the uptake of the findings in the community [2, 19].

If health research is meant to improve health outcomes of all individuals, the historical absence of women from health research is potentially detrimental to women’s health [2, 19–21]. CBR offers a critique of traditional biomedical research approaches where patriarchy and sexism have prevailed and potentiates an opportunity to address the knowledge gap related to women’s health [1]. As such, academics in the field of women’s health have started to engage in CBR and other forms of unconventional, participatory research [22–26].

In the field of HIV, the systematic exclusion of women from clinical studies has been particularly problematic [19]. Given the rapid medical advances in HIV, the exclusion of women has yielded significant issues in their HIV care. Epidemiological HIV data of women remains relatively sparse despite the feminization of HIV; clinical understandings of women regarding dosage and toxicity to antiretroviral therapies are limited due to such treatments being tested in trials with a predominance of male participants and women-focused research is almost non-existent [19]. Due to the gendered realities of HIV, women living with HIV often possess unique care needs that go overlooked [27]. Furthermore, when insufficiently included, women’s unique considerations are rarely elicited [19]. These circumstances of unique need and community informant capacity in terms of study development potentiate an ideal situation for the use of CBR [28]. The aim of this paper is to share our research team’s experience with the process of initiating, developing, and implementing a large national longitudinal cohort study involving women with HIV using a CBR approach. It is our intention that we demonstrate to other quantitative health researchers that a CBR approach is feasible and beneficial within large health research projects.

### Description of CHIWOS

The Canadian HIV Women’s Sexual and Reproductive Health Cohort Study (CHIWOS) is a national, multi-site, inter-disciplinary, CBR, quantitative, longitudinal cohort study that seeks to understand whether and how women-centred HIV care (WCHC) [28] may improve health outcomes for women living with HIV in Canada. Cohort data collection for CHIWOS was launched in 2013 in British Columbia (BC), Ontario (ON), and Quebec (QC), with electronic, peer research associate (PRA)-driven data collection. PRAs are women with HIV who are hired and trained to conduct research; in this case: the recruitment, consenting and survey administration. As of May 1, 2015 1425 women with HIV were enrolled in CHIWOS and had completed the baseline interview questionnaire. Two additional time points are scheduled at 18 and 36 months (a complete description of CHIWOS can be found at www.chiwos.ca).

**Fig. 1:**
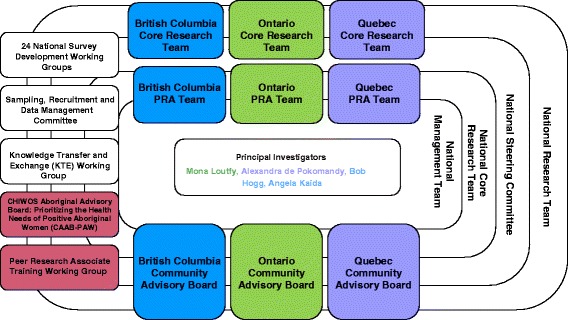
The CHIWOS Study Team Structure. A diagram illustrating the community-based research (CBR) team structure used by the CHIWOS Study Team. Developed during the formative phase of CHIWOS to ensure meaningful involvement of all stakeholders. The boxes identify the various groups and committees involved in the management of CHIWOS and the overarching structure of the Study Team

CHIWOS is working towards a flexible, transformative and action-oriented approach to women’s health research. CHIWOS’s goals are to address a gap in knowledge related to women and HIV in Canada from the perspective of women. The specific aims of CHIWOS are to estimate: 1) the proportion and patterns of, as well as the factors associated with, WCHC uptake, and 2) the effect of WCHC on their overall (quality of life), HIV [e.g., antiretroviral therapy (ART) use, viral suppression], women’s (e.g., cervical and breast cancer screening), mental (e.g., depression), sexual (e.g., sexual satisfaction), and reproductive (e.g., contraceptive use, pregnancy) health outcomes among women with HIV in Canada. CHIWOS has brought together a national, multi-disciplinary research team, drawing expertise and experience from various disciplines (Fig. 1). In addition to our CBR approach, CHIWOS is guided by critical feminist and social justice frameworks and considers social determinants of health and intersectionality perspective across the lifespan [29–31].

## Discussion

Our team, including women with HIV, identified early on that in order to create meaningful knowledge on the health and care of women with HIV, a comprehensive understanding of community experiences and needs was essential and thus the vital need to use a CBR approach emerged. As our experience with CBR has evolved, we have been tasked with reflecting on how the study team has operationalized CBR principles, our successes, and our challenges from both academic and community lenses. Our joint perspective creates a comprehensive dialogue about the value of CBR, as we have formed a team with significant diversity, from clinicians to epidemiologists, research assistants to PRAs.

Conceptually, CHIWOS was born out of pursuing topics based on community identified needs and the potential impact for improving care for women with HIV. The newly formed CHIWOS team began the project by brainstorming and developing a study vision, mission, mandate and values (Table 1), all of which were led and informed by community expertise. Table 2 presents our theoretical and research frameworks and guiding principles, which include critical feminism, anti-oppression, intersectionality and social justice [29–31]. This was followed by a two-year long formative phase, which included qualitative data collection with women from across the country to inform our understanding of WCHC. The CHIWOS team then embarked on a year-long CBR survey development process described elsewhere [32]. Community members, including trained PRAs and other women with HIV, were asked to pilot and revise the survey. After piloting the draft questionnaire, PRAs and participants were asked to describe their experience, and explain any concerns related to the survey. The crucial community feedback garnered from these discussions informed subsequent revisions, including: cutting sections that felt redundant, unjustifiably intrusive, or excessively long; rephrasing questions to better reflect the needs, understandings, and identities of women; and adjusting skip patterns to ensure the relevance of questions to particularly situated women. These insights were essential to informing the development of the final survey and, ultimately, the involvement of a wide inter-disciplinary team.

**Table 1:** CHIWOS Vision, Mission, Mandate & Core Values

CHIWOS Vision, Mission, Mandate & Core Values		
Vision, Mission and Mandate	Vision	CHIWOS envisions a country where all women living with HIV are able to achieve optimal health and well-being, no matter where they are in their experience of HIV and in their lives. CHIWOS aims to contribute to this vision through transformational women-centred community-based research and action that is driven by HIV-positive women, researchers, care providers and policy makers, in all of their diversity, together, within an equitable and mutually respectful environment.
	Mission	CHIWOS is committed to creating new knowledge that will be used to support women living with HIV in Canada to achieve optimal health and wellbeing through meaningfully involving them in every stage of the research process by providing a safe, innovative, and transformational research environment.
	Mandate	To assess the barriers to and facilitators of women-centred HIV care use and the impact of such patterns of use on overall, HIV, mental, women’s, sexual and reproductive health outcomes of women living with HIV across Canada, through excellence in women-centred community-based research.
Core Values	Integrity	CHIWOS believes that integrity should be at the core of everything we do. Integrity is the quality of being honest and responsible. It is the willingness to act according to the ethics, values, beliefs and principles that we hold as members of CHIWOS.
	Respect	CHIWOS strives to promote feelings of esteem and interact in such a way as to promote that esteem among all members. This means having a sense of the worth or excellence of oneself and others, both as professionals and human beings. It also means behaving in ways that would bring credit and honour upon oneself and the team to which one belongs.
	Accountability	CHIWOS encourages its members to accept responsibility for their actions and work. It is hoped that members of this project will see themselves accountable to each other as well as to women living with HIV in Canada.
	Inclusivity	CHIWOS acknowledges the multiple, complex, and overlapping identities that create a rich and vibrant community with many different experiences of health and wellness. All of these experiences will be shared and honoured.
	Equity	CHIWOS understands that disparities in health result from systemic inequalities that are unjust and unfair, and will work to address these disparities holistically.
	Partnership and Collaboration	CHIWOS is committed to working in partnership with community members, and other stakeholders in HIV-positive women’s health, at all stages of our research. Diverse forms of knowledge are valued and inform our work. Collaboration deepens and strengthens our impact.
	Empowerment	CHIWOS strives to create a forum for the celebration of existing capacities and skills, and to create an opportunity to build on the skills, abilities and the courage of individuals and communities to make informed choices, and to transform those choices into desired actions and outcomes.
	Social Action	CHIWOS aims to be transformational. The research process and the knowledge produced will act as vehicles for positive and sustainable social change that will promote health and wellness among women living with HIV.

**Table 2:** Theoretical and Research Approaches & Guiding Principles

CHIWOS Theoretical & Research Approaches & Guiding Principles		CHIWOS definition	Operationalization (completed/in progress/planned)
Theoretical approaches	Women-centred Community-based Research	- Involves the community of interest, women living with HIV, at all stages of research: developing research questions, research procedures and questionnaire; carrying out research; data analysis, and result dissemination. - Appreciates the different expertise of community members and academic researchers and has them both being equal partners as part of the project. - Understands the involvement of all different types of women and women at different stages of their life. - Understands that women’s lives are busy and tries to accommodate their different needs with flexibility.	- Women living with HIV are involved as part of the national Steering Committee, provincial Community Advisory Boards (CABs) and Peer Research Associates (PRAs) in developing research questions and the questionnaire, as well as all other aspects of the study, including recruitment, troubleshooting data quality and interview challenges, knowledge translation and exchange (KTE), etc. - PRAs are involved on various management committees and working groups; research decisions include input from the PRAs. - Women living with HIV at various stages of life have been hired as PRAs. - Flexible among all teams members, including working with PRAs schedules, holding PRA and team meetings in the afternoon for those with children and in the evening for those with jobs.
	Critical Feminist Approach	- Analyzes the impact of structural inequalities, and gender-based marginalization and oppression. - Holds gender as socially situated, complex, and non-binary. - Understands the other diverse aspects of identity, power, and reality that shape individuals and communities experiences; recognizes how sexism, racism, classism, ableism, homophobia, transphobia, HIV-related stigma, and other axes of oppression intersect.	- Both CHIWOS’s process and its outcome goals are centred around principles of anti-oppression, an analysis of structures of power, and a vision of being transformative and action-driven. - It is a community-based project which seeks to create a new research community of doctors, care providers, academics, students, activists, community members, and other stakeholders, driven by mutual respect and goals of social change. - All involved receive training with a “What is CHIWOS?” presentation (available at www.chiwos.ca) and discussion of critical feminist and anti-oppression approaches. - The team partakes in ongoing discussions of reflexivity regarding power, anti-oppression amongst any other issue that is raised.
	Intersectionality	- Analyzes the overlapping and intersecting nature of identities and oppressions that shape individual lives and experiences, and an acknowledgement of the mutually supportive and constitutive nature of hierarchies and structures of power along different axes. Also acknowledges and values how identities and communities can be sites for resistance, resilience, and support.	- CHIWOS is attentive towards the diverse social positioning of the community of women. Recognizing the many intersecting identities that women living with HIV inhabit, we have tried to develop a research instrument that reflects the needs of trans women, Aboriginal women, immigrant women, women of colour, queer women, and other communities/experiences such as those of women with children, women involved in sex work, young women, etc. (recognizing that none of the groups are mutually exclusive). We have worked throughout the development process with stakeholder groups able to focus on different community needs to work towards a tool that is acceptable and respectful for the diversity of the women living with HIV community.
Research approaches	Anti-Oppression and Anti-Racism	- Related principles of anti-oppression further emphasize that women within and between societies are positioned differently and are differently impacted by the complexities of privilege and power relations.	- Maintain an analysis of oppression (sexism, racism, etc.) as central to our research goals and instruments as we try to capture the impact of racism and other forms of oppression on women’s experiences, but also to our process – for example, integrating an anti-oppression workshop into PRA training.
	Social Determinants of Health	- Our project understands that social factors have key implications on health and that simply administering medical treatment is often insufficient to improve health. Poverty, gender inequity, and a multitude of other factors have a major impact on women’s vulnerability to health problems and (in)ability to access care and support.	- We developed the survey instrument with a keen eye towards capturing the many non-medical factors that impact women’s health status and care. In the formative phase focus groups discussions, we were sure to explicitly ask about structural barriers to care in order to capture people’s experience with some of these social determinants of health.
Guiding principles	Social Justice and Human Rights	- The research holds to emancipatory goals of creating meaningful change in affected communities, and to challenge oppression, improve health, eliminate barriers, and further social justice.	- We are engaged in action-driven research, setting out to assess the need for change to existing care and service provision models, and inform that change to better meet the needs of women living with HIV. We recognize that health disparities and unjust barriers to accessing good health/care are deeply entrenched and tied into large structures of oppression, but hope that the results of this work will contribute in meaningful ways to improving care for women living with HIV.
	Meaningful Involvement of Women living with HIV/AIDS & Greater Involvement of Persons living with HIV/AIDS	- Women living with HIV must be recognized as equal partners in all stages of the project. All contributions and experiences are respected and valued; women living with HIV are recognized as experts in their own lives. These principles demand the intent to foster self-determination and agency in the community, and should be engaged in without tokenism.	- We assembled CABs in each province to bring together diverse perspectives and experience from women living with HIV. - In order to ensure strong community voices, there is a PRA from each province on the National Management Team and Steering Committee, and PRAs lead other Working Groups. - We worked closely with PRAs throughout development and implementation of the survey instrument through an extensive and iterative community-based consultative process, including women living with HIV and continue to do so in the new surveys.
	Ownership, Control, Access, and Possession (OCAP™)	Definitions from First Nations Centre (2005): Ownership, control, access, and possession, or OCAP™, is self-determination applied to research. It is a political response to tenacious colonial approaches to research and information management. *Ownership*: Ownership refers to the relationship of a First Nations community to its cultural knowledge/data/information. The principle states that a community or group owns information collectively in the same way that an individual owns their personal information. *Control*: The aspirations and rights of First Nations Peoples to maintain and regain control of all aspects of their lives and institutions extend to research, information and data. The principle of control asserts that First Nations Peoples, their communities and representative bodies are within their rights in seeking to control all aspects of research and information management processes which impact them. First Nations control of research can include all stages of a particular research project – from conception to completion. The principle extends to the control of resources and review processes, the formulation of conceptual frameworks, data management and so on. *Access:* First Nations Peoples must have access to information and data about themselves and their communities, regardless of where it is currently held. The principle also refers to the right of First Nations communities and organizations to manage and make decisions regarding access to their collective information. This may be achieved in practice through standardized, formal protocols. *Possession:* While ownership identifies the relationship between a people and their data in principle, possession or stewardship is more literal. Although not a condition of ownership per se, possession (of data) is a mechanism by which ownership can be asserted and protected. When data owned by one party is in the possession of another, there is a risk of breech or misuse. This is particularly important when trust is lacking between the owner and possessor.	- Recognizing the significant impact of HIV among First Nations communities in Canada, and the violent legacy and ongoing colonialism, CHIWOS is committed to working towards OCAP™ principles and building an ongoing relationship with First Nations communities. - The creation of the CHIWOS Aboriginal Advisory Board: Prioritizing the Health Needs of Positive Aboriginal Women (CAAB-PAW) was a crucial step in working towards this in part, by reviewing and informing CHIWOS’s work, and to ensure CHIWOS strives to accomplish its goals for and with Aboriginal women.

A critical aspect of the CBR process was to work with and train PRAs, and importantly, to provide them with the support necessary to ensure their ongoing involvement in the project. A team of 8-28 PRAs was hired and trained in each province. A national team developed the curriculum for the training based on principles of participatory adult learning and the insights of modules developed for other studies. Provincial trainings were organized over several days to build relationships amongst the team members; review important concepts; discuss the CHIWOS project and its approach, research principles, and team structure; provide practical training around recruiting and obtaining consent from potential participants, administering the online survey instrument, accessing the supports available to PRAs, and other relevant information. An anonymous evaluation was solicited from the PRAs to inform subsequent trainings and ongoing learning opportunities. A secure online platform for PRA training and networking was also created for refresher training and newly hired PRAs. We also created a process through which PRAs connect on a regular basis with each other and provincial coordinators and investigators to provide input and receive updates, usually through monthly teleconference. The support provided to each PRA needed to be tailored to the unique needs of the individual. Understanding the needs of each PRA occurred over time and policies were developed and recorded in order to ensure that these strategies were upheld.

PRAs experienced challenges due to the varying expectations of what their new role would encompass. Consequently, the team had to be innovative in ensuring each PRA felt that their contributions were manageable and meaningful depending on each PRAs capacities and interests. In light of this, new opportunities were developed for the PRAs to be involved in the project in a variety of leadership capacities. Some of these positions include being National Management Team and Knowledge Translation and Exchange Working Group PRA Representatives. We have also learned that meaningful involvement and adequate support for PRAs must include appropriate compensation, recognition and acknowledgement. For any voluntary commitment there is no pressure or expectation that PRAs attend. This process has been challenging because, ideally, PRAs should be compensated for all of their work. The consequence of not being able to financially afford this ideal presents challenges to acknowledging all contributions.

Through the unique role of being a PRA, many women continue to experience the challenge of “wearing many hats”. On several occasions, PRAs have completed interviews with friends, family members or clients. Furthermore, they may be perceived and treated differently by women in the community based on their new PRA position within CHIWOS. These experiences also tie into the challenge of setting appropriate boundaries with participants and navigating the thin line that exists between these varying relationships due to the multiple roles of the PRA. CHIWOS has developed “Challenging Scenario Guidelines” to support PRAs experiencing challenges brought to the forefront throughout the project.

Despite these ups and downs, the CHIWOS PRAs are the heart of this project. In partnership with various clinical and community sites in each province, PRAs have led the national cohort data collection phase. Word of mouth, recruiting from their personal and professional networks and utilizing peer-driven recruitment strategies have been the most successful strategies. The community connections, experiences, and aptitudes of our PRAs have also enabled successful recruitment from many under-served and harder-to-reach communities. Consequently, our cohort is inclusive of trans people, women who have experience with sex work, First Nations, Métis and Inuit women, women with a history of incarceration, African, Caribbean and Black women, and women not accessing care.

While our successes can feasibly be implemented by future research teams where CBR is well suited, the challenges that we have encountered raise important considerations for how to resolve, or at least attempt to resolve, the tensions that ultimately emerge when taking a CBR approach to national research. A key tension that we continue to struggle with is the reality that our consultative process requires more time. This is poorly understood and accepted at a bureaucratic level. We have also experienced issues with PRA compensation at a bureaucratic level. These two challenges capture the team’s overall experience of navigating CBR within large academic settings that are unfamiliar with a CBR process. In consideration of planning a large-scale CBR project, it would be advisable to meet with leadership within your institution to ensure they understand and will fully support the CBR process.

Because CHIWOS was developed as a CBR project with a specific emphasis on equity and anti-oppressive approaches to research, power imbalances, such as decision-making roles, also occurred providing the research team with the opportunity to reflect and find solutions aimed to maximize decision-making equitability. The most effective solution was expanding communication. Establishing an understanding in the formative phase of each team member’s role, contributions, communication style, and skills was also particularly vital to the sustainability and success of this large, national CBR guided project.

Given the predominant quantitative expertise among the research team, there were also challenges in shifting toward a CBR approach that needed to be openly discussed. This entailed, and will continue to entail, ongoing reflective discussions that work toward identifying possible methodological and ethical tensions that often emerge when doing CBR that relies on multiple forms of knowledge [33]. These tensions are not easily resolvable, but our reflective attention to them reinforces and supports our accountability to enact the long list of critical feminist principles and core values that guide us in our research.

## Conclusions

We believe that our reflections on our process of using CBR can generally be applicable to any health research studies; however, we acknowledge that the clinical and social complexity of HIV may result in some unique realities of CBR. CHIWOS offers new expertise on how to reframe health research approaches to women and HIV in keeping with the belief that research has the potential to transform the lives of communities through active engagement. We advocate that by academics, community members and participants sharing with and learning from each other, we can strengthen and develop important frameworks, principles and practices aimed at integrating the complex process of CBR into medical research. As others have previously stated, we also suggest that health research needs to be action-oriented, and not just undertaken simply for the sake of knowledge production [34]. Research can be re-conceptualized as a process, not just an outcome. CBR challenges us to consider how this process itself can be an agent of change through partnerships and capacity building [16].

## Abbreviations

CHIWOS, Canadian HIV Women’s Sexual and Reproductive Health Cohort Study; ICWNA, International Community of Women living with HIV, North America; HIV/AIDS, Human Immunodeficiency Virus/Acquired Immunodeficiency Syndrome; PRAs, peer research associates; CBR, community-based research; CIHR, Canadian Institutes of Health Research; OHTN, Ontario HIV Treatment Network; NIH, National Institutes of Health; WCBR, women’s HIV CBR; NCRT, National Core Research Team; CABs, community advisory boards; GIPA/MIPA, greater and meaningful involvement of people living with HIV; MIWA, meaningful involvement of women living with HIV; BC, British Columbia; QC, Quebec; KTE, knowledge translation and exchange; CTN, Canadian HIV Trials Network; AHSC, Academic Health Science Centres; AFP, alternative funding plans; OCAP, ownership, control, access, and possession; CAAB-PAW, CHIWOS aboriginal advisory board-positive aboriginal women
